# Towards Lead-Free Piezoceramics: Facing a Synthesis Challenge

**DOI:** 10.3390/ma9010021

**Published:** 2016-01-02

**Authors:** María Elena Villafuerte-Castrejón, Emilio Morán, Armando Reyes-Montero, Rodrigo Vivar-Ocampo, Jesús-Alejandro Peña-Jiménez, Salvador-Oliver Rea-López, Lorena Pardo

**Affiliations:** 1 Instituto de Investigaciones en Materiales, Universidad Nacional Autónoma de México, Ciudad Universitaria, Circuito Exterior S/N, A.P, México D.F. 70-360, Mexico; ingaremo@gmail.com (A.R.-M.); ocampovivar@gmail.com (R.V.-O.); jesusalejandropea@gmail.com (J.-A.P.-J.); salvatore_0389@hotmail.com (S.-O.R.-L.); 2 Departamento de Química Inorgánica, Facultad de Ciencias Químicas, Universidad Complutense, Madrid 28040, Spain; emoran@quim.ucm.es; 3 Instituto de Ciencia de Materiales de Madrid (ICMM), CSIC, Sor Juana Inés de la Cruz, 3. Cantoblanco, Madrid 28049, Spain; lpardo@icmm.csic.es

**Keywords:** synthesis, sintering, lead-free, BT, BCZT, BNT, KNN, ceramics, ferroelectricity, piezoelectricity

## Abstract

The search for electroceramic materials with enhanced ferro-pyro-piezoelectric properties and revealing the perovskite type structure has been the objective of a significant number of manuscripts reported in the literature. This has been usually carried out by proposing the synthesis and processing of new compounds and solid solution series. In this work, several methods to obtain ferro-pyro-piezoelectric families of materials featuring the well-known ABO_3_ perovskite structure (or related) such as BaTiO_3_, Ba_1–*x*_Ca*_x_*Ti_1–*y*_Zr*_y_*O_3_, (Bi_0.5_Na_0.5_)TiO_3_, (K_0.5_Na_0.5_)NbO_3_ and their solid solutions with different cations either in the A or B positions, are presented. For this kind of materials, the challenge for obtaining a single phase compound with a specific grain size and morphology and, most importantly, with the adequate stoichiometry, will also be discussed. The results reviewed herein will be discussed in terms of the tendency of working with softer conditions, *i.e.*, lower temperature and shorter reaction times, also referred to as soft-chemistry.

## 1. Introduction

**Figure 1:**
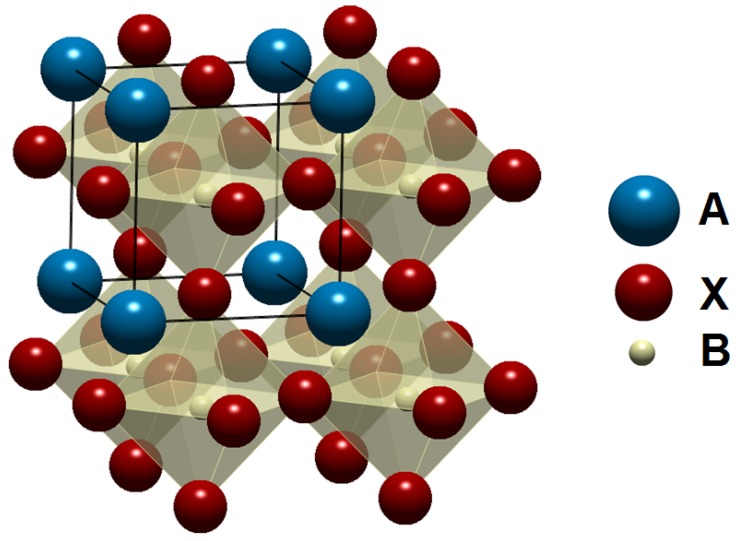
Cubic prototype perovskite structure. Usual cations in lead-free piezoceramics: A = Ba^2+^, Ca^2+^, Bi^3+^, Na^+^K^+^; B = Ti^4+^, Zr^4+^, Nb^5+^; X = O^2−^.

Ferroelectrics with perovskite type structure (Figure 1) are important materials used to produce piezoelectric ceramics for applications in sensors, actuators, motors, resonators, *etc.* [1,2,3]. The most widely used piezoelectric ceramics for those applications are those based on the lead titanate zirconate *x*PbTiO_3_-(1−*x*)PbZrO_3_ solid solution, also known as PZT, because of the advantages derived from their good properties and the wide possibilities for modifying their structure and properties by numerous dopants [4,5]. During the last two decades [6], and due to the increasing importance of environmental protection, due to the high toxicity of lead and lead oxide, a great number of countries in all continents have legislated to replace this material, which sparked intense research on lead-free piezoelectric ceramics. Some objectives of industrial transference of these materials have already been achieved, whereas some basic problems remain unsolved [7]. In analogy to the characteristics of the PZT phase diagram, which presents a Morphotropic Phase Boundary (MPB), between tetragonal and rhombohedral phases, where the electromechanical properties exhibit an improvement, a lot of studies have been made in order to find MPB in different ceramic systems [8,9,10,11,12,13].

Worth noting, from a chemical point of view, the synthesis and processing of these materials is not simple and some challenges are yet to be faced. Stoichiometric control, search for MPB, softer synthesis conditions, adequate grain size, adequate ferroelectric domain size and distribution, sintering conditions, and scalability and cost for industrial transfer are among the main challenges.

First of all, stoichiometric control is difficult to achieve when volatile species, such as Bismuth or alkaline metal oxides, are involved. To solve this problem, softer conditions of synthesis are needed and in this connection wet chemistry methods, such as sol-gel [14], Pechini [15,16], hydrothermal [17,18], *etc.* are useful. Worth mentioning, microwave assisted methods are nowadays gaining interest because they offer many advantages and are amenable to industrial transfer [19,20,21]. Obviously, the complexity of many of the most interesting compositions makes it quite difficult to obtain single phase, homogeneous materials and careful examination of the corresponding phase diagrams is necessary to design the synthesis routes.

Moreover, as stated above, MPB are crucial for obtaining piezoelectric properties and searching for similar MPB, as PZT is a key issue in finding suitable new compositions. Structural and microstructural characterization near the MPB composition is a delicate task due to the coexistence of crystal symmetries [9].

Regarding the enhancement of the electromechanical properties, a lot of work has been done looking for nanomaterials [22]. Nevertheless, nano-sized grained ceramics do not present the optimal piezoelectric properties. This is due to the difficulty in the application of a DC electric field to align the spontaneous polarization, the so-called poling process. Poling is needed to induce piezoelectricity in the ceramics. However, properly sintered nano-sized powder results on fine-grain and dense ceramics. Due to their homogeneous microstructure [23], they have good piezoelectric performance. In this regard, microwave sintering can be achieved at lower temperatures in shorter times, which promotes fine-grain.

The performance of ceramics, from an application point of view, is evaluated through different parameters. In this work, we will consider the following parameters: *d*_ij_(piezoelectric charge constant), which indicates the ratio of the output strain (*j*-axis) under an electric field input (*i*-axis); and *k*_ij_ (electromechanical coupling factor), which shows the efficiency of the material in the conversion of electrical energy into mechanical energy. The factor *k*_p_ expresses coupling for the planar resonance of a thin disk of ceramic, thickness poled. Dielectric permittivity, ɛ^T^_ij_, and dielectric dissipation factor (or loss tangent, tan δ) are also parameters of interest, currently measured at 1 kHz. One of the main properties of ferroelectrics is the temperature dependence of the permittivity that increases up to a maximum value (lambda curve) at the phase transition to the high temperature paraelectric, non-polar, phase. The Curie temperature (*T*_C_) is calculated from the inverse of the permittivity (1/ɛ^T^_ij_) *vs.* temperature curve. *T*_C_ is the intersection with the *T*-axis of the linear fit of (1/ɛ^T^_ij_) above the transition. The piezoelectric performance vanishes at the transition temperature, which limits the range of applications of the material.

There are lead-free ferroelectrics with higher spontaneous polarization than lead-titanate (>100 μC/cm^2^). We can mention BiFeO_3_ [24] among others [25], which are very promising for use as piezoelectrics. A number of problems remain to be solved for industrial applications (e.g., the control of the electrical resistivity or special conditions of stability).

Other interesting materials are the bismuth layered structured ferroelectrics (BSLF), highly competitive with PZT at high temperature (>200 °C), but with poor performance at room temperature [26].

Among the lead free piezoelectric materials studied in the last years, four groups will be analyzed here: BaTiO_3_ (BT), Ba_1−*x*_Ca*_x_*Ti_1−*y*_Zr*_y_*O_3_ (BCZT), (Bi_0.5_Na_0.5_)TiO_3_ (BNT) and (K_0.5_Na_0.5_)NbO_3_ (KNN), because their properties are already competitive with those of PZT for similar applications. A number of recent reviews in the literature [27,28,29] and a book [30] focused on their properties can be recommended to the reader.

## 2. BaTiO_3_ (BT)

**Figure 2:**
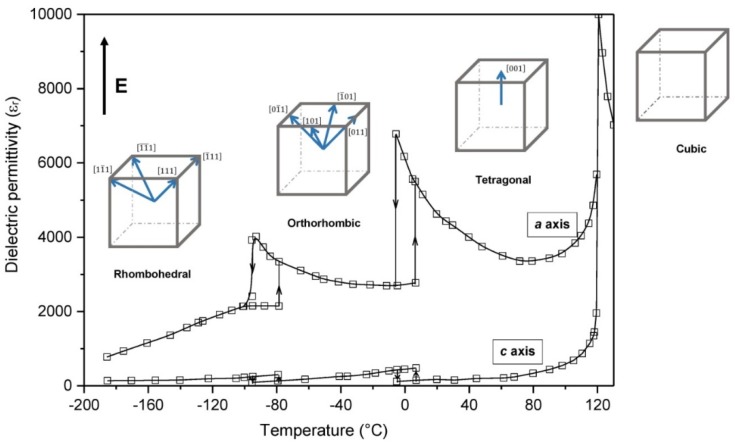
Phase transition temperatures at the dielectric permittivity curves of BaTiO_3_ and equivalent polar directions under an applied electric field (*E*) for the polymorphic phases related to the cubic prototype perovskite structure.

Barium titanate, the first ferro-piezoelectric oxide with perovskite structure, has been continuously studied and used since the 1940s when discovered [31] within the context of Second World War; actually, it was the first ferroelectric material without hydrogen bonding [32]. It is an archetypical electroceramic widely used in the capacitors industry for its exceptional properties: very high dielectric constant, high quality factors and low temperature coefficient of the dielectric constant. Worth noting, several structural phase transitions (Figure 2) occur when changing the temperature: cubic (above 120–135 °C), tetragonal (between 120 °C and 125 °C), orthorhombic (between 5 °C and −90 °C) and rhombohedral (below −90 °C). Because of the high symmetry, the cubic phase of barium titanate exhibits paraelectricity and an isotropic dielectricity albeit with a high dielectric constant. Below the Curie temperature (120–135 °C), the crystal structure transforms to the distorted tetragonal which causes a spontaneous polarization and a ferroelectric and piezoelectric behavior [33,34]. However, the low Curie temperature reduces the range of applications. Regarding its piezoelectric properties, it has a high electromechanical coupling factor, *k*_33_ ≈ 0.50, and piezoelectric strain constant, *d*_33_ ≈ 190 pC/N and, among other applications, it has been used in sonars. Nevertheless, the performances as piezoelectric material are well below those of PZT, and this has been the main obstacle for wider applications as actuators and sensors. Nowadays, because BT is the simplest lead-free piezoelectric ceramic, many data are available and some new piezoelectric materials present a similar crystal structure, much work is being carried out. New methods of synthesis are tested in order to improve BT piezoelectric performance. Much of this effort is made looking for nanocrystalline BT powders which will be further sintered and the results so far obtained indicate that BT (or related) ceramics possess high potential for being used as lead-free piezoelectric materials [7].

**Figure 3:**
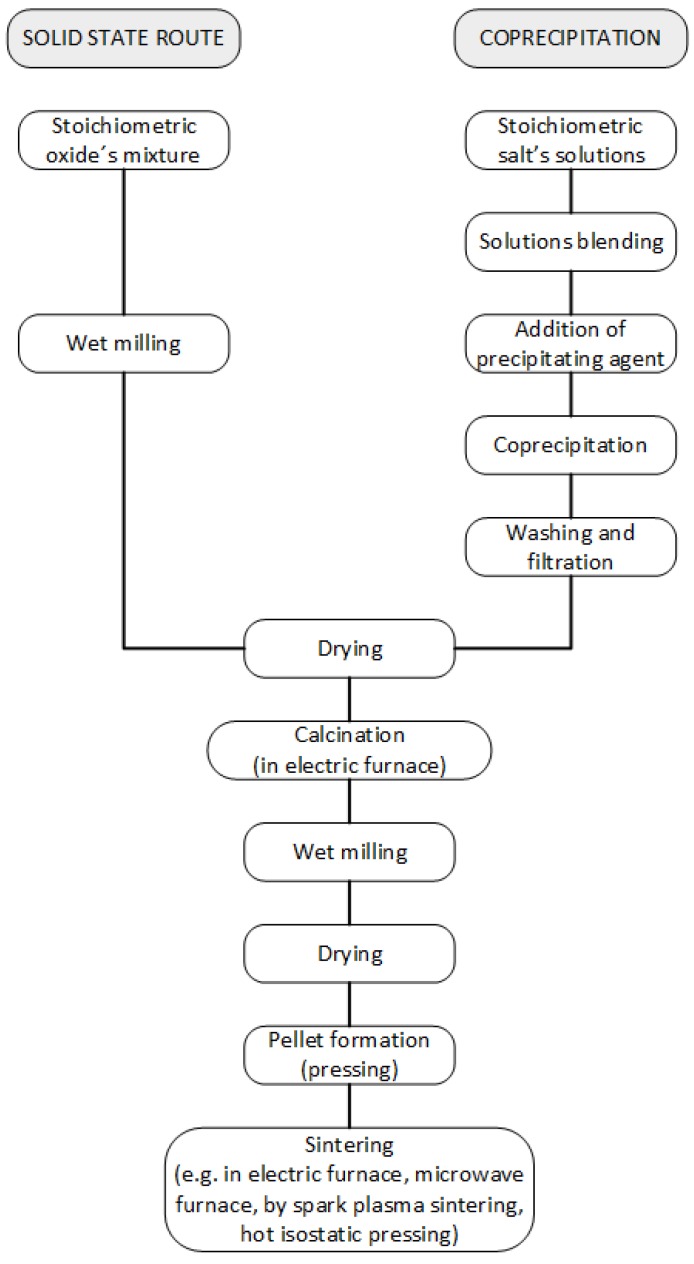
Flux diagram of the conventional route for processing piezoelectric ceramics and alternative coprecipitation route.

The most commonly used method for BT synthesis is the conventional ceramic route (Figure 3) using BaCO_3_ and TiO_2_ as raw materials which are heated at 600–800 °C for 1–2 h and sintered at 900–1350 °C for 2–4 h in air [8]. This method is simple and easy: the reactants must be carefully mixed and high temperature must be applied in order to obtain a homogenous powder.

These high temperature conditions provoke grain growth; the resulting microstructure has a wide grain-size distribution, some degree of porosity is always present, and secondary phases, such as Ba_2_TiO_4_ and others, may be formed. Thus, although widely used, the ceramic route has many drawbacks and BT ceramics are not competitive for piezoelectric applications. However, recently, as an example of careful work, high piezoelectric properties and adequate domain configuration in BaTiO_3_ ceramics obtained through the solid-state reaction route have been reported by Shao, *et al.* [35]. These authors report a significant improvement of the piezoelectric properties of BT obtained by the solid state method (1130 °C for 4 h.), as a function of the sintering temperature and grain size, the best results (*d*_33_ = 419 pC/N; *k*_p_ = 0.453 and tanδ = 1.36% at room temperature) being obtained at a sintering temperature of 1210 °C with average domain width in the poled BT of about 240 nm and a decrease of the grain size down to 2μm.

**Figure 4:**
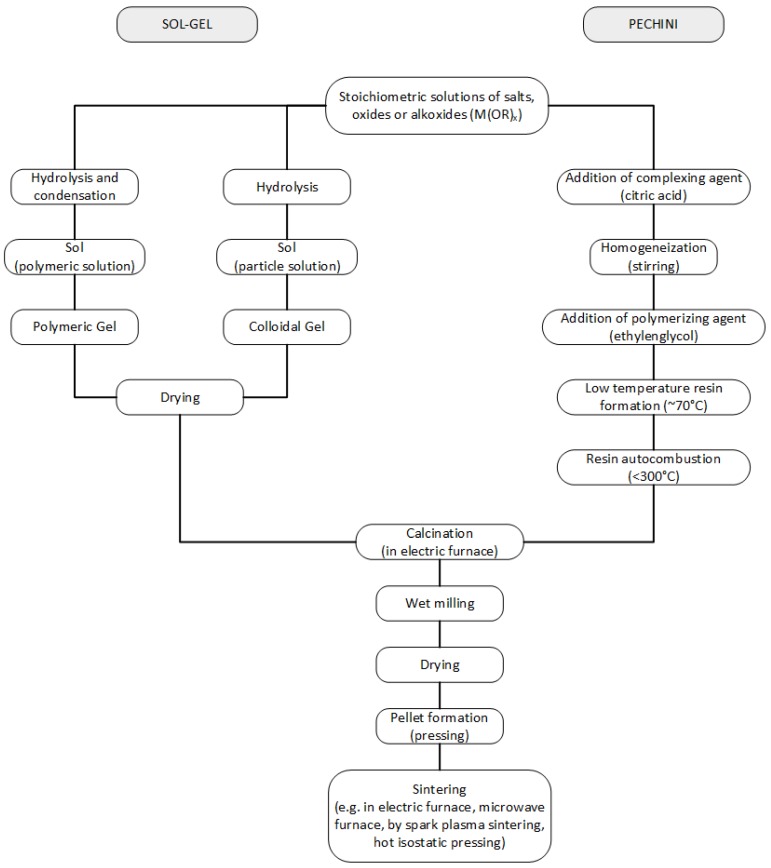
Flux diagram for sol-gel and Pechini synthesis methods.

**Figure 5:**
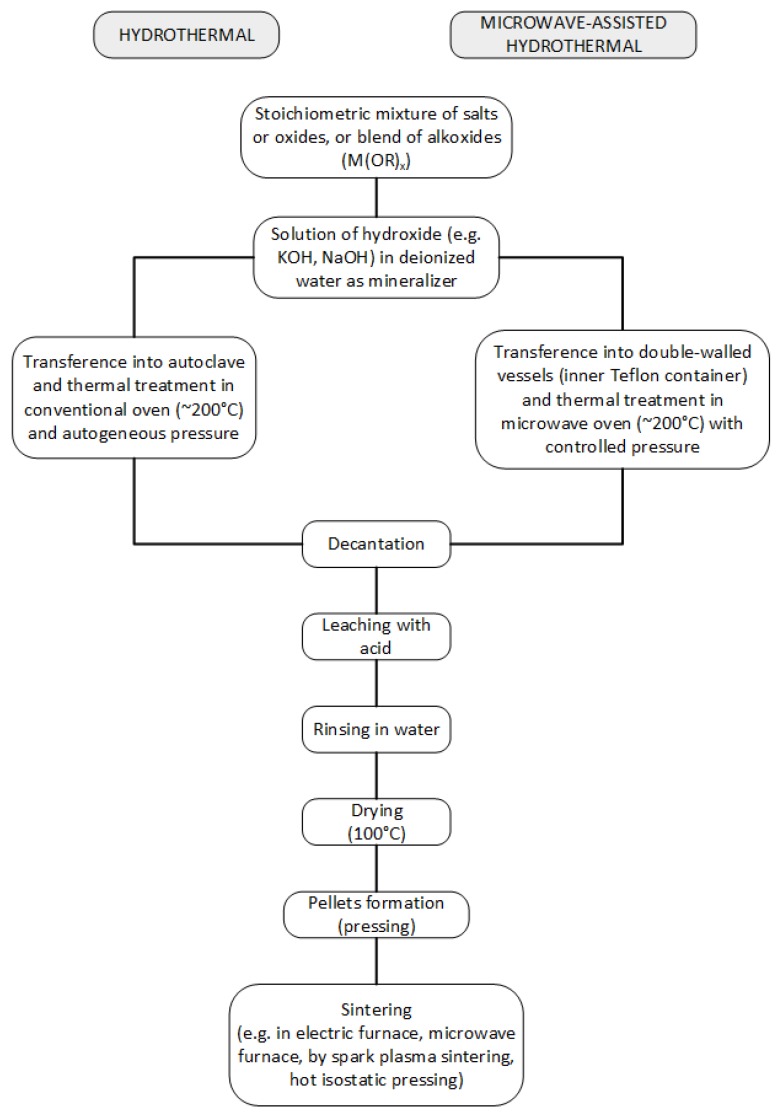
Flux diagram for hydrothermal and microwave assisted hydrothermal synthesis methods.

Owing to the above mentioned drawbacks of the ceramic method, a great effort is being devoted to the development of new methods [36,37]. Noteworthy, alternative, lower temperature synthetic methods such as coprecipitation (Figure 3), sol-gel, Pechini (Figure 4), hydrothermal, microwave-assisted (Figure 5), *etc.*, are being explored aiming at the preparation of high purity nano-powders with controlled stoichiometry and better sinterability and, consequently, better and reproducible quality. Once the BT submicron or nano-powders are obtained, sintering conditions are an important issue which strongly affects the properties as they correlate with the final grain and ferroelectric domain sizes [38,39]. Starting with the sol-gel processes, wet synthesis routes, using different precursors such as acetate, stearate [40], nitrates [41] and oxide [42] for barium and titanium (IV) alkoxides (*i.e.*, Ti-isopropoxide) as the source for titanium are employed; organic alcohols are the reaction media [43]. It is recognized that the sol-gel method is complex and involves several steps—hydrolysis, condensation, drying and calcination—and the final properties will be affected by changes made during the entire process (*i.e*., concentrations, pH, temperature, time, *etc.*). A common challenge for this kind of sol-gel methodology is the agglomeration of nanoparticles, and some authors claim that, by using surfactants such as oleic acid, this problem can be overcome and tetragonal BT can be obtained with room temperature permittivity higher than 3000 and 98.5% density [44]. Precipitation methods from aqueous solutions of inorganic compounds such as precursors Ba(OH)_2_ and TiCl_4_ can also be used and, interestingly, the concentration of Ba^2+^ ions has a strong influence on the particles homogeneity and crystallite size [45,46].

Some authors report on the kinetics and mechanism of this direct reaction [47]. In this connection, an ambient condition sol process (ACS), which produces nanocrystalline cubic phase has been reported [48]. With a different approach but the same goal, an electrochemical route has been explored, using Ti metal plate in KOH and Ba(OH)_2_ aqueous electrolyte with absolute ethanol, nanoparticles of the cubic phase being obtained [49]. The permittivity and the dielectric loss values, measured as a function of the sintering temperature change, for BaTiO_3_ synthesized from various salts (NO_3_^−^, Cl^−^ and acetates), has been reported, with the highest permittivity value being found for samples prepared from Cl^−^ salts, with the smallest particle size and a 1250 °C sintering temperature. Although, the role of the anions is not well understood. Since the search for lead-free ceramics is driven by environmental concerns, a comment on the various levels of toxicity of the precursors in these methods must be added here and, consequently, all mentioned routes cannot be considered in the same manner.

Another wet method with considerable success is the hydrothermal synthesis (Figure 5) due to its ability to provide highly pure and crystalline powders. Actually, hydrothermally made BT is already of commercial use and some companies such as Sakai Chemical Industry Co., Ltd. (Osaka, Japan) and Toda Industry (Ho Chi Minh, Vietnam) provide high quality powders of 0.1 μm grain size. Hydrothermal synthesis is carried out in closed vessels (usually made in stainless steel and lined with Teflon) at temperatures between 80 °C and 240 °C and with autogenous pressure [17]. Therefore, the method is a low temperature, low energy one, and it is “environmentally benign” since the synthesis is accomplished in a closed system from which different chemicals can be recovered and recycled. Nonetheless, working in sealed conditions provides a CO_2_-free environment, which avoids BaCO_3_ formation, a common impurity. For the synthesis of BT, barium hydroxide or soluble barium salts are used as the source of barium and anatase, or amorphous TiO_2_^−^ hydrated gels are used as the source of titanium in the presence of concentrated hydroxide ion; mixed precursors such as Ba-Ti acetate can also be used. These procedures usually result in submicron cubic BT—the paraelectric form—at room temperature which need to be heated afterwards at higher temperatures of 800–1100 °C to obtain the ferroelectric tetragonal form. Hydrothermal synthesis, when performed at low temperatures (95 °C) using BaCl_2_ and anatase in basic NaOH solution, always yields the cubic form while working at higher temperatures (240 °C) with BaCl_2_ and hydrous titanium oxide (prepared by hydrolyzing titanium isopropoxide with hydrochloric acid) resulting in the tetragonal form. In this regard, some authors conclude that the powder obtained from BaCl_2_ and TiCl_4_, with 80 nm particle size and 40% tetragonal content, has the highest dielectric permittivity (6200) and the lowest dielectric loss [49]. Interestingly enough, it seems that hydrothermally made powders of BaTiO_3_ may contain large amounts of protons in the oxygen sublattice and these defects are compensated by vacancies on metal sites; by annealing the powder at *T* < 600 °C water is released and the point defects disappear [18].

A variation of this method, the so-called microwave-assisted hydrothermal synthesis is becoming increasingly used since it has several advantages such as significantly lowering reaction times (minutes or hours rather than days), uniform nucleation of the powders in suspension, energy and cost efficiency among others [50]. Worth mentioning is the pioneering work that uses this methodology for a plethora of inorganic compounds, among them the barium titanate [19]: the microwave assisted hydrothermal synthesis (MW-HS) of BT was done at 138 °C with reaction time as low as 15 min, using barium nitrate, titanium chloride and KOH as starting chemicals [21,51]. Several works using laboratory-made microwave heating autoclaves (or domestic ovens) working at the usual frequency of 2.45 GHz were reported [52,53]. Nowadays, microwave furnaces with pressure, temperature and power control can be used. As starting materials Ba(OH)_2_ Ba(NO_3_)_2_, BaCl_2_, *etc.* can be used as source for barium and TiOCl_2_, TiCl_4_, *etc.*, for titanium; different equimolar or slightly higher concentrated Ba^2+^ solutions are employed. The most common mineralizer used is KOH in different concentrations. Depending of the conditions, particles ranging from 0.1–0.5 and submicron-sized equi-axed BaTiO_3_ particles with monosized distribution are obtained [54,55,56,57]. Worth mentioning, microwaves can also be employed for sintering the ceramic powders previously obtained and pelletized.

The sintering process plays an important role in the BaTiO_3_ properties and many works have reported the influence of the conventional sintering process and the microwave sintering on the materials properties. As it is well-known, microwaves interact readily with polar species producing heat and this is the case of dielectric materials such as BT which will absorb the radiation in a very efficient way. As many authors point out, it is difficult to prepare BaTiO_3_ dense ceramics via the conventional sintering process even if nano-powders are used [57]. The nano-sized particles have the tendency to form agglomerates that cause defects on the microstructure. In samples prepared by the Pechini route, a two-step sintering method, first at 1300 °C, then cooled down and held at 1100 °C for 0–20 h, results in much greater dielectric permittivity than that of the normal sintering method [57]. The reported piezoelectric properties changed with the synthesis method from 190 pC/N, obtained by the conventional solid state route to 350 pC/N in samples synthesized by hydrothermal method and afterwards microwave sintering at 1300 °C [58]. The authors attributed the high *d*_33_ value to the sintering density (98.3%), the appropriate grain size: 3.1–3.4 μm and the high dielectric constant over 4000. Worth noting, the same authors correlate the excellent piezoelectric performance of the microwave sintered BT with the microstructure (grain boundaries and domains size) as observed by different microscopic techniques: the size of the ferroelectric domains is less than 50 nm and the fraction of random boundaries is approximately 10% higher than that of conventionally sintered samples. This work has been followed by more recent papers using microwaves both for the synthesis and the sinterization (at 1150 °C/30 min.) with similar piezoelectric properties [59,60].

To conclude this part we can state that barium titanate BT, which piezoelectric properties are not competitive with in comparison to PZT, is now coming under focus since it is a lead-free ferro-piezoelectric, the properties of which are being improved by using alternative methods of synthesis, many of which need lower temperatures and yield nanomaterials. The adequate sintering of these nanomaterials results in improved piezoelectric properties. Worth noting is the use of microwave-assisted methods (both for synthesis and sintering), and, last but not least, all the work performed in barium titanate, could in principle, be extended to BT-derived materials, such as the solid solutions between Bi_0.5_Na_0.5_TiO_3_ and BaTiO_3_ and others.

## 3. BaTiO_3_-BaZrO_3_-CaTiO_3_ (BCZT)

**Figure 6:**
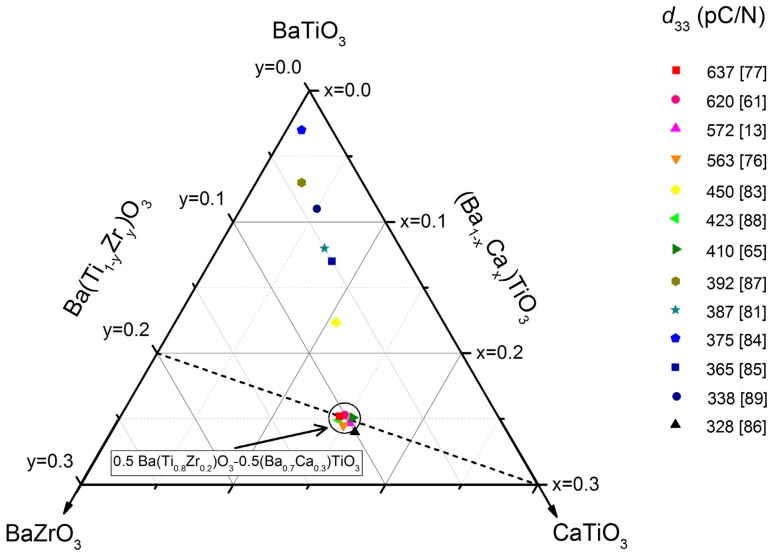
Ternary diagram for the system BaTiO_3_-BaZrO_3_-CaTiO_3_. Piezoelectric *d*_33_ coefficient values for studied compositions are shown. The corresponding references are also shown in brackets.

To enhance the piezoelectric and dielectric properties, many BaTiO_3_-based solid solutions with different A-site and B-site dopants (where, typically, A = Ca, Sr, La; B = Nb, Ta, Zr) are used. Especially Ca^2+^ and Zr^4+^ doping, hereinafter called Ba_1−*x*_Ca*_x_*Ti_1−*y*_Zr*_y_*O_3_ , have been the focus of numerous publications after a high piezoelectric coefficient (*d*_33_ = 620 pC/N) was reported for Ba_0.85_Ca_0.15_Ti_0.90_Zr_0.10_O_3_, *i.e.*, within the pseudo-binary solid solution system (1−*w*)Ba(Ti_0.80_Zr_0.20_)O_3_-*w*(Ba_0.70_ Ca_0.30_)TiO_3_ for *w* = 0.50 [61] (Figure 6). This composition stays at the vicinity of a tricritical point of this system, in the frontier of the high temperature cubic non-ferroelectric phase, with the two ferroelectric ones, rhombohedral and tetragonal phases. It has been shown that cubic phase coexists with the two ferroelectric ones, greatly enhancing the polarizability of the ceramic. The low *T*_C_ ≈ 93 °C, high synthesis (1300 °C for 2 h) and sintering (1450 °C for 3 h) conditions, not suitable for industrial production, and the subsequently large grain structures are among the drawbacks that exist in this composition. Microstructures with average grain size > 10 µm obtained to date are not optimized for the present trend of device miniaturization. This feature is therefore important in order to increase the frequency range of the ultrasonic transduction. Therefore, strategies to reduce the synthesis and sintering temperatures are needed for processing routes towards sub-10 µm grain size ceramics. Most of the work has been carried out following the solid state synthesis route, and the role of dopants and substitutions [14,15,61,62,63], the processing related ceramic microstructure [64,65,66,67], resulting in strong changes in the piezoelectric coefficient for the same composition (Figure 6), and the poling process [68] have been the current topics of this research line.

As for the BaTiO_3_-CaTiO_3_ binary system, the addition of Ca^2+^ into the site A of BaTiO_3_ increases the stability temperature range of the tetragonal phase [69] by shifting to lower values the ferroelectric(FE)-rhombohedral (R) to the FE-orthorhombic (O) (T_1_) and the FE-orthorhombic (O) to the FE-tetragonal (T) (T_2_) FE-FE phase transition temperatures. Following the same strategy, it was recently shown that an amount of 0.06 < *x* < 0.10, enhances the dielectric and piezoelectric properties of the Ba(Ti_0.94_Sn_0.03_Zr_0.03_)O_3_ lead-free ceramics [70] without the drawback of reducing the Curie temperature (*T*_C_) of the FE-tetragonal (T) to paraelectric (PE)-cubic transition.

As for the BaTiO_3_-BaZrO_3_ binary system, the substitution of Zr^4+^ into the B site produces an undesired reduction of *T*c, while T_1_ and T_2_ increase at different rates. For *y* = 0.15, three ferroelectric phases (R, O and T) coexist near room temperature. It was expected at the pinched point a great polarizability, similar to the one at the morphotropic phase boundary of the PZT; however, no ferroelectric and piezoelectric improvement was achieved. This was probably due to the degradation of ferroelectricity. Furthermore, the ceramic shows broad dielectric peaks with frequency dispersion, *i.e.*, ferroelectric relaxor behavior with increasing Zr^4+^ concentration (*y* > 0.25) [71,72].

Some works have been focused on increasing *T*_C_ (up to 114 °C) at the expense of the piezoelectric performance (less than 450 pC/N) by changing the composition to (1−*w*)Ba(Zr_0.15_Ti_0.85_)O_3_-*w*(Ba_0.7_Ca_0.3_)TiO_3_ with *w* = 0.53 (*i.e*., Ba_0.84_Ca_0.16_Ti_0.93_Zr_0.07_O_3_), and still sintering at 1450 °C for 3 h [73]. In order to reduce processing temperatures and times, using conventional ceramic technology, one option is to add CeO_2_ [74] or MnO [75]. Also, LiF, Ga_2_O_3_ and Y_2_O_3_ have been added to BCZT ceramics with sintering temperatures ranging from 1350–1500 °C and *d*_33_ over 360–440 pC/N were obtained.

Microstructural effects in the properties were also examined in Ba_0.85_Ca_0.15_Zr_0.1_Ti_0.9_O_3_ ceramics processed by wet chemistry [76]. The average grain size associated with this samples was 30 μm accompanied by higher domain density mostly with 90° twinning *d*_33_ as high as 563 pC/N were obtained. However, in order to avoid the mechanical instability associated with high grain size, in a recent work [65] it was possible to obtain Ba_1__−_*_x_*Ca*_x_*Ti_0.9_Zr_0.1_O_3_ (*x* = 0.10, 0.15) ceramics by solid-state synthesis using a reduced treatment at 1250 °C (2 h) and different sintering temperatures (1300, 1350, 1400 °C for 2 h) to minimize the grain size (<10 μm) while keeping good piezoelectric properties (*d*_33_ = 410 pC/N).

A recent work [63] compares the properties of BCZT obtained by solid state reaction and by hydrothermal synthesis route followed by microwave sintering. The compounds synthesized by the solid state method showed high permittivity, while those prepared by the hydrothermal route exhibited an improvement in their ferroelectric and piezoelectric properties. The hydrothermal samples showed a relatively smaller and homogeneous grain size (∼100 nm) than the solid-state samples (∼800 nm). Also, a sol-gel method was proposed [14], but no properties were reported in this work. However, the best *d*_33_ have been recently obtained in ceramics processed by sol-gel synthesis [77].

As an alternative to conventional solid state synthesis and in order to reduce the processing temperatures, the Pechini method has been recently used to obtain BCTZ ceramic materials [15]. This technique is based on having individual cations complexed with poly-functional organic acids (citric acid is preferred) and a polyhydroxyl alcohol (commonly ethylenglycol). The effectiveness of this method is maintaining ions mixed at atomic level when they form a polymer resin after heating around 80–110 °C. This resin can be calcined at low temperature (500–800 °C) to produce very fine powders with narrow crystal size distribution. Dense ceramics (95%–98% theoretical density) were obtained [16] using low sintering conditions (1200 °C, 1250 °C, 1275 °C for 5 h) with *T*_C_ = 118 °C. The best piezoelectric properties were obtained for the ceramic sintered at 1275 °C (*d*_33_ = 390 pC/N, *d*_31_ = 143 pC/N, *k*_p_ = 50%).

The sintering parameters are important factors which determine the grain size and then the compound properties: temperature, dwell time [78], soaking time [79], and process [80]. Several authors proposed an optimal sintering temperature at 1400–1450 °C for obtain better piezoelectric properties, W. Li reports a BCZT with *d*_33_ = 387 pC/N [81]. Wu, *et al.* obtained a *d*_33_ = 442 pC/N and *k*_p_ = 48.9% in a BCZT ceramic sintered at 1440 °C, with improved electrical properties in the MPB composition, with the coexistence of rhombohedral and tetragonal phases [82]. Some authors [73] compared three sintering methods: spark plasma, two-step and normal sintering to obtain dense ceramics with different grain sizes. With grain size up to more than 10 µm, samples exhibit excellent piezoelectric properties: *d*_33_ > 470 pC/N and *k*_p_ > 0.48, whereas in samples with grain size of 0.4 µm, the piezoelectric properties are rather poor: *d*_33_ ~ 72pC/N, *k*_p_ ~ 0.13. Alternatively, a microwave sintering on the samples prepared by the two different methods mentioned above [63] was proposed, the combination of hydrothermal synthesis and microwave sintering gave samples with better dielectric relaxation, ferroelectric properties and *d*_33_, while the permittivity is lower.

The sintering process and temperature determine the grain size and then the material properties. Poling conditions (electric field and the temperature) must be taken into account. An enhanced piezoelectric behavior of *d_33_* = 637 pC/N, *k*_p_ = 0.56 is obtained for the Ba_0.85_Ca_0.15_Ti_0.90_Zr_0.10_O_3_ ceramics synthesized by sol-gel when poled at 40 °C with 40 kV/cm field [77]. Table 1 summarizes the mentioned and some other relevant works [83,84,85,86,87,88,89] published on this solid solution series. The synthesis route and sintering temperatures as well the properties *T*_C_, ε**^T^_33_**, *d*_33_ and *k*_p_ and the grain sizes are shown.

**Table 1:** Some results of the publications in the system BaTiO_3_-BaZrO_3_-CaTiO_3_.

Composition (%)		Synthesis Method	Synthesis (*T (t)*) (°C (h))	Sintering (*T (t)*) (°C (h))	Grain Size (μm)	ε^T^_33_/tan δ (%) (at RT)	*T*_C_ (°C)	*d*_33_/*k*_p_ (pCN^−1^/%)	Reference
Ba(Ti_0.8_Zr_0.2_)O_3_-*x*(Ba_0.7_Ca_0.3_)TiO_3_	*x* = 0.00−1.00	Solid State	1350 (–)	1450–1500 (–)	–	2500–3060/–	20–80	200–620/–	[61]
Ba(Zr_0.15_Ti_0.85_)O_3_-*x*(Ba_0.8_Ca_0.2_)TiO_3_	*x* = 0.00−1.00	Solid State	1300 (2)	1450 (3)	10–30	1000–2250/–	70–120	450–600/–	[83]
(Ba_1−*x*_Ca*_x_*)(Ti_0.98_Zr_0.02_)O_3_	*x* = 0.00−0.04	Solid State	1200 (4)	1450 (4)	–	1100–2200/–	110–120	270–375/44–32	[84]
(Ba_1−*x*_Ca*_x_*)(Ti_0.95_Zr_0.05_)O_3_	*x* = 0.02−0.20	Solid State	1200 (4)	1450 (4)	–	1800–2900/–	100–110	200–365/32–48	[85]
(Ba_1−*x*_Ca*_x_*)(Ti_0.90_Zr_0.10_)O_3_	*x* = 0.12−0.18	Solid State	1200 (4)	1450 (4)	5–15	3000–4800/–	60–70	240–328/30–38	[86]
(Ba_0.93_Ca_0.07_)(Ti_0.95_Zr_0.05_)O_3_	–	Solid State	1200 (4)	1300–1500 (4)	10	1000–2300/–	105–115	142–387/26–44	[81]
(Ba_1−*x*_Ca*_x_*)(Ti_0.04_Zr_0.96_)O_3_	*x* = 0.00−0.09	Solid State	1200 (4)	1300, 1400 (4)	24–39	1700–2000/2	123–126	158–392/–	[87]
(Ba_0.85_Ca_0.15_)(Ti_1__−__x_Zr_x_)O_3_	*x* =0.00−0.20	Solid State	1200 (3)	1500 (2)	10–30	2000–3000/1.5	70–130	50–423/20–49	[88]
(Ba_1__−_*_x_*Ca*_x_*)(Zr_0.1_Ti_0.9_)O_3_	0.00 ≤ *x* ≤ 0.20	Solid State	1200 (6)	1400–1420 (4)	–	1000–3000/–	80–90	160–550/30–58	[13]
(Ba_0.85_Ca_0.15_)(Zr*_y_*Ti_1__−_*_y_*)O_3_	0.00 ≤ *y* ≤ 0.15	Solid State	1200 (6)	1400–1420 (4)	–	1000–4000/–	60–120	140–580/35–58	[13]
(Ba_0.95_Ca_0.05_)(Zr*_y_*Ti_1__−_*_y_*)O_3_	0.00 ≤ *y* ≤ 0.15	Solid State	1200 (6)	1350 (2)	9–14	1200–2000/1–3	60–125	175–340/16–35	[89]
Ba_1__−_*_x_*Ca*_x_*Ti_0.9_Zr_0.1_O_3_	*x* = 0.10, 0.15	Solid State	1250 (2)	1300–1400 (2)	0.5–10	2800–3400/1.2	75–80	175–410/22–50	[65]
(Ba_0.95_Ca_0.05_)(Ti_0.85_Zr_0.15_)O_3_	–	Pechini	700 (4)	1100–1300 (2)	–	–	–	–	[15]
Ba_0.9_Ca_0.1_Ti_0.9_Zr_0.1_O_3_	–	Pechini	700 (1)	1200–1275 (5)	0.4–15.0	2200–2500/1.2	115	45, 240, 390/5, 49, 50	[16]
Ba_0.85_Ca_0.15_Ti_0.9_Zr_0.1_O_3_	–	Sol-gel	800 (5)	1200–1500 (10)	0.5–32.0	800–2900/1–5	85–902	21–563/–	[76]
(1−*w*)Ba(Ti_0.80_Zr_0.20_)O_3_−*w*(Ba_0.70_ Ca_0.30_) TiO_3_	0.48≤ *w* ≤ 0.52	Sol-gel	600–1000 (4)	1200–1500 (2)	14–20	–	–	400–637	[77]

## 4. (Bi_0.5_Na_0.5_)TiO_3_-BaTiO_3_ (BNT-BT)

Bi_0.5_Na_0.5_TiO_3_ (BNT) has been known as ferroelectric since 1961 [90], and BNT-based ceramics [91,92] are among the most promising lead-free piezoelectric materials to compete in actuator applications with PZT [93] BNT shows ferroelectric properties with a relatively large remnant polarization, *P*_r_ = 38 mC/cm^2^, a relatively large coercive field, *E*_C_ = 73 kV/cm and Curie temperature of 320 °C [90]. Classically, it was considered that BNT crystallizes with a perovskite-like structure with a sequence of phase transitions on-cooling from cubic (C) to high-temperature tetragonal (T) (actually an ergodic relaxor) to room-temperature ferroelectric rhombohedral (R) structures. The high- temperature T and R phases appear to coexist over a rather broad (≈250 °C) temperature range [94]. Nevertheless, at sintering temperatures [95], the Bi and Na are volatiles with the consequent variation in the stoichiometry besides the poling difficulty due to its large coercive field and relatively large conductivity, thus preventing its use as a piezoceramic.

Therefore, a big effort was made in studying solid-solution systems to overcome these difficulties in the use of BNT ceramics. Piezoelectric properties of modified BNT based materials were expected to be enhanced when solid solutions have a morphotropic phase boundary (MPB) [4]. Typically [91] for the solid solution with BaTiO_3_ ((1−*x*)BNT-*x*BT or BNBT100*x*) at room temperature, the MPB separates rhombohedral and tetragonal ferroelectric phases. However, the MPB differs strongly with respect to that of PZT. It presents a wide range of compositions 0.05 < *x* < 0.11 that at room temperature are cubic in the absence of an electric field [96] and undergo a phase transition to ferroelectric phases under the action of the electric field [97]. Similar effects were observed in other BNT based solid solutions [93].

Several BNT-based binary and ternary systems with MPB compositions with different cations has been proposed (BNT-ATiO_3_ (A = Ca, Sr, Ba and Pb), BNT-KNbO_3_, BNT-Bi_0.5_Li_0.5_TiO_3_, BNT-Bi_0.5_K_0.5_TiO_3_(BNT-BKT), BNT-K_0.5_Na_0.5_NbO_3_(BNT-KNN), BNT-BKT-KNN, BNT-BT-KNN, BNT-BKT-BiFeO_3_, BNT-BKT-BaTiO_3_-SrTiO_3_, *etc.*) [98,99].

Among these systems, the solid solution BNT-BT has been widely studied [91,92,93,99]. Particularly, when poled, the ceramics with compositions close to BNBT6 were considered at the Morphotropic Phase Boundary (MPB), between rhombohedral and tetragonal ferroelectric phases at room temperature. Typically, these BNBT6 ceramics show an electromechanical coupling factor for thickness resonance of thin disks of *k*_p_ = 52% and a *d*_33_ = 125 pC/N. These are not very high values in comparison with other lead-free ceramics of compositions already reviewed here, but they will be used similarly to compare the ceramic performance obtained from different synthesis methods. Worth noticing, the transition from the unpoled cubic phase to the ferroelectric phases at the MPB gives these BNBT ceramics at the MPB unique properties as actuators [90].

**Figure 7:**
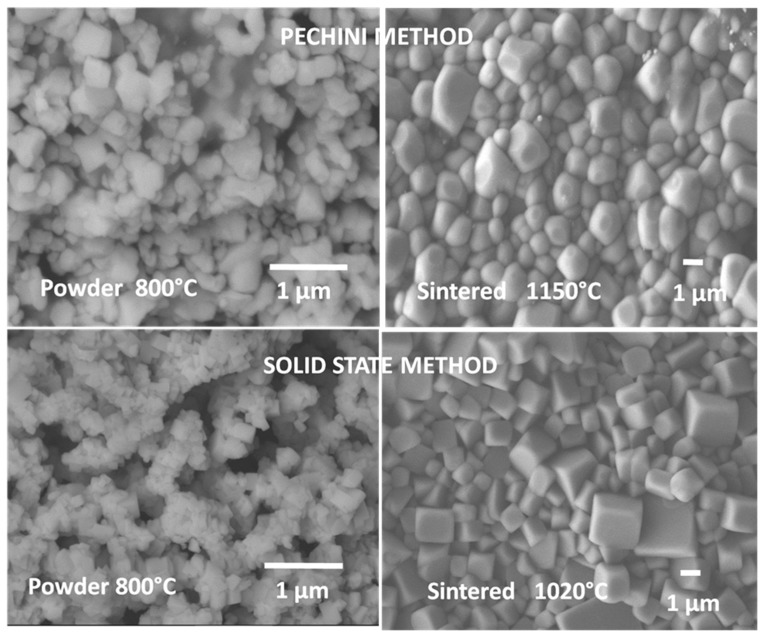
SEM micrographs of synthesized powder (by solid state route and Pechini method) and sintered ceramics of (1-*x*)Bi_0.5_Na_0.5_TiO_3_-*x*Ba_0.9_Ca_0.1_TiO_3_ (*x* = 0.06) composition. Note the influence of the powder particle morphology on the ceramics’ microstructure.

BNT based compositions can be prepared by various methods. The conventional solid state reaction implies high temperatures and, as it was mentioned before, the volatility of the cations is hard to avoid and detrimental to properties [100]. Besides the difficulty in maintaining chemical homogeneity, the particles obtained are poorly uniform with broad size distributions. Some recent works proposed the synthesis of BTN based powders by various wet chemical routes (Figure 7), such as the hydrothermal method [98,101,102,103], Sol-gel [104,105,106,107] and emulsion [108] among others. Some of the mentioned works on sol-gel routes use the so-called “citrate method”, which is very similar to the Pechini (Figure 4) but without using ethylenglycol as a polymerizing agent. The reason is that the citric acid, besides being a chelating agent, also plays a polymerizing role.

A summary of the abovementioned and some other relevant works [106,107,108,109,110,111,112,113,114,115,116] published on BNT based ceramics is shown in Table 2, as well as some of their properties. References [106] and [115] use the so-called combustion methods, combined with sol-gel [106] or using urea as an ignition promoter [115]. These are fast chemistry routes in which, currently, synthesis takes place within minutes. From this table the influence of the synthesis method on the properties of the studied compounds can be seen, being the highest *d*_33_ values those obtained in compounds synthesized by wet chemistry methods at lower temperatures and also those near the MPB. The effect of the additives as La, Nd, Ce, Nb, Co, Mn also have influence on their properties, those obtaining the highest *d*_33_ were with Mn, Nd, and the solids solutions (Bi_0.47_Na_0.47_Ba_0.06_TiO_3_)_0.99_(Ba_0.77_Ca_0.23_TiO_3_)_0.01_ and (Bi_0.5_Na_0.5_TiO_3_)_0.95_-[(Ba(Zr_0.2_Ti_0.8_)O_3_) (Ba_0.7_Ca_0.3_TiO_3_)]_0.05_.

Even if there are still a number of open questions about this composition due to structural complexity and relaxor properties arising from a large coexistence of polymorphs [94], BNBT near MPB is considered a good candidate for lead-free piezoelectric ceramics due to the properties reported in the early 90s [91] and recent understanding of the peculiarities of the MPBs [93].

**Table 2:** Some results of the publications on compositions based on (Bi_0.5_Na_0.5_)TiO_3_-BaTiO_3_.

Composition (%)	Synthesis Method	Synthesis (*T* *(t)*) (°C (h))	Sintering (*T* *(t)*) (°C (h))	Grain Size (μm)	ε^T^_33_/tanδ (%) (at RT)	*T*_C_ (°C)	*d*_33_/*k*_p_ (pCN^−1^/%)	Reference
Bi_0.5_Na_0.5_TiO_3_	Solid State	800 (5)	1100 (5)	4.1	425/3.2	–	94.8/–	[100]
Bi_0.5_Na_0.5_TiO_3_	Hydrothermal	700 (4)	1010 (2)	8.6	324/2.14	–	77.3/15.9	[109]
Bi_0.5_Na_0.5_TiO_3_	Hydrothermal	200 (12)	–	2	–	–	–	[102]
Bi_0.5_Na_0.5_TiO_3_	Hydrothermal	200 (72)	–	0.04–0.15	–	–	–	[101]
Bi_0.5_Na_0.5_TiO_3_	Microwave-assisted hydrothermal	180 (8)	–	0.04–0.15	–	–	–	[101]
Bi_0.5_Na_0.5_TiO_3_	Hydrothermal	150–200 (5–20)	–	0.05–0.2	–	–	–	[103]
Bi_0.5_Na_0.5_TiO_3_	Sol-gel	600–850 (2)	1000–1200 (2)	<0.2	–	–	–	[104]
(Bi_0.5_Na_0.5_)_0.95_Ba_0.05_TiO_3_	Citrate method	700 (4)	1050 (–)	1	–	–	–	[105]
(Bi_0.5_Na_0.5_)_0.05_Ba_0.95_TiO_3_	Solid State	850 (4)	1200 (2)	–	2211/17.5	126	78/–	[110]
(Bi_0.5_Na_0.5_)_0.93_Ba_0.07_TiO_3_	Solid State	850 (4)	1150–1250 (2–4)	1–2	–/–	246	148/15	[111]
(Bi_0.5_Na_0.5_)_0.93_Ba_0.07_TiO_3_	Citrate method	600 (1)	1150 (2)	–	–/–	–	176/21.2	[112]
(Bi_0.5_Na_0.5_)_0.94_Ba_0.06_TiO_3_	Solid State	800 (1)	1200 (2)	–	580/1.3	288	125/19	[91]
(Bi_0.5_Na_0.5_)_0.94_Ba_0.06_TiO_3_	Solid State	1000 (2)	1160–1200 (2)	–	776/2.5	–	117/28	[12]
(Bi_0.5_Na_0.5_)_0.94_Ba_0.06_TiO_3_	Combustion	500 (few mins)	1100 (2)	–	698/–	–	125/27.2	[106]
(Bi_0.5_Na_0.5_)_0.94_Ba_0.06_TiO_3_	Emulsion	700 (3)	1200 (3)	1–2.5	–/–	–	174/28	[108]
(Bi_0.5_Na_0.5_)_0.94_Ba_0.06_TiO_3_	Mechanosynthesis	800–900 (2)	1160–1180 (1)	–	–/1.7	–	122/29	[92]
(Bi_0.5_Na_0.5_)_0.94_Ba_0.06_TiO_3_	Citrate method	600(1)	1150 (2)	–	600/5	–	180/28	[113]
(Bi_0.5_Na_0.5_)_0.94_Ba_0.06_TiO_3_ + 3 mol % Bi_2_O_3_	Solid State	800( 2)	1150–1200 (2)	–	–/2.65	–	165/24	[114]
(Bi_0.5_Na_0.5_)_0.94_Ba_0.06_TiO_3_ + 0.25 at % Eu	Combustion	600 (few mins)	1150 (4)	–	1658/–	–	149/5.5	[115]
(Bi_0.5_Na_0.5_)_0.94_Ba_0.06_TiO_3_ + La	Solid State	1000 (2)	1160–1200 (2)	–	1576/4.5	–	125/24	[12]
(Bi_0.5_Na_0.5_)_0.94_Ba_0.06_TiO_3_ + Co	Solid State	1000 (2)	1160–1200 (2)	–	1200/2.3	–	139/27	[12]
(Bi_0.5_Na_0.5_)_0.94_Ba_0.06_TiO_3_ + 0.8 wt % Nd_2_O_3_	Solid State	800 (–)	1150 (2)	2	1947/5.7	–	175/31	[116]
(Bi_0.5_Na_0.5_)_0.91_(Ba_0.07_Ca_0.03_)_0.09_TiO_3_	Solid State	850 (3)	1170 (2)	–	–/–	–	125/33	[98]
(Bi_0.47_Na_0.47_Ba_0.06_TiO_3_)_0.99_-(Ba_0.77_Ca_0.23_TiO_3_)_0.01_	Solid State	850 (2)	1100–1200 (2)	–	–/–	–	178/27	[117]

## 5. (K_0.5_Na_0.5_)NbO_3_ (KNN)

Among lead-free ceramics, potassium-sodium niobate, with general formula (K*_x_*Na_1−*x*_)NbO_3_, is considered an interesting material (for *x* values close to 0.5) because of its high Curie temperature and good piezo and ferroelectric properties. Actually, piezoelectric materials based on KNN were the first to be widely studied in the search for lead-free piezoceramics due to environmental considerations [6,118,119,120], and this solid solution system is still considered as the best global alternative to PZT for practical applications [7,121]. Prior to further considerations, the interested reader is also referred to a recently published comprehensive and excellent review (including 640 references) [29].

KNN is a solid solution between two relevant materials featuring the perovskite structure: KNbO_3_ (ferroelectric) and NaNbO_3_ (antiferroelectric); both showing orthorhombic symmetry at room temperature; their respective Curie temperatures (associated to tetragonal-cubic phase transitions) are as high as 435 °C and 355 °C, respectively. In fact, these ferroelectric perovskites were discovered in the 1950s and 1960s, single crystals were grown and their properties thoroughly investigated, but research on alkali niobates in ceramic form was not considered until a few years later [122,123,124], when the difficulty of sintering these compositions and the need to use expensive or complex procedures to prevent the occurrence of exaggerated grain growth in the production of mechanically stable ceramics were revealed.

As regarding structure, KNN shows a perovskite structure, orthorhombic at room temperature, with a space group *Amm2,* different from those of the end members of the solid solution: KNbO_3_ changes from rhombohedral to orthorhombic symmetry at −10 °C and from this to tetragonal at 225 °C; NaNbO_3_ shows orthorhombic symmetry (space group *Pbcm*). KNN presents three phase boundaries corresponding to approximate values of *x* = 0.17, 0.35 and 0.5, [125], but most of the studies are concentrated around the *x* = 0.5 value since this composition, close to a morphotropic phase boundary (MPB) separating two orthorhombic phases, exhibits a moderate dielectric constant and an optimum piezoelectric response. The phase diagram is quite complicated in comparison with the well-known PZT one [1].

KNN presents three phase boundaries corresponding to approximate values of *x* = 0.17, 0.35 and 0.5, but most of the studies are concentrated around the *x* = 0.5 value since this composition, close to a morphotropic phase boundary (MPB) separating two orthorhombic phases, exhibits a moderate dielectric constant and an optimum piezoelectric response [121].

However, pure KNN ceramics present some drawbacks such as a poor densification and off-stoichiometry induced by volatilization during sintering. Actually, soft-chemistry routes succeeded in preparing phase-pure KNN. Fine powders were synthesized via a solid-state route from a homogeneous solid mixture. A colloidal dispersion comprising a mixed ethanol solution of potassium and sodium acetates and Nb_2_O_5_ fine particles was attrition milled and dried carefully to avoid water absorption. Ceramics were finally obtained by two-step calcination in air at 450 °C and 625°C, each for 3 h [126]. Also, pure KNN ceramics with improved piezoelectric properties (*d*_33_ = 80–120 pC/N) were obtained by using sintering aids to reduce sintering temperatures to 1000 °C for 2 h [127].

**Figure 8:**
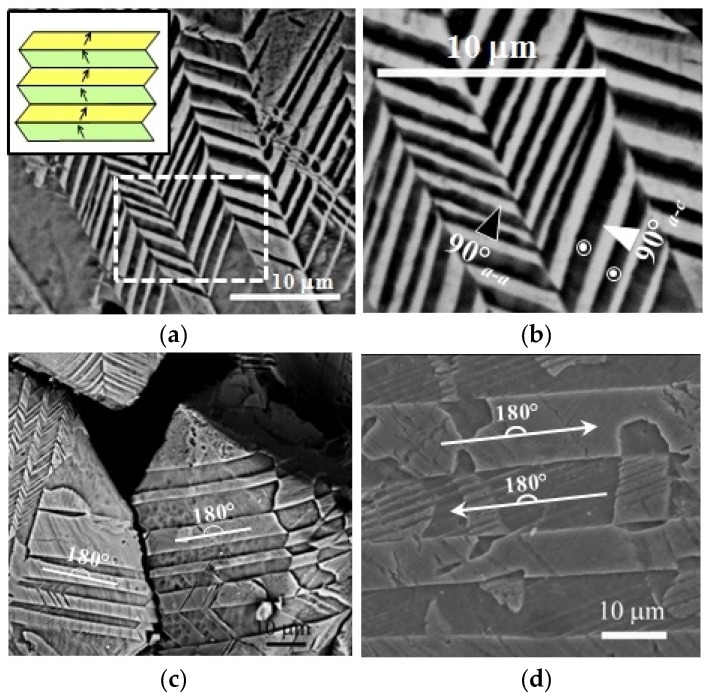
KNN samples prepared by microwave-assisted hydrothermal synthesis. Representative microstructure showing (**a**) lamellar 90° a-a; and (**b**) dagger-shaped 90° a-c domains. The inset shows a scheme of the 90° wall twin structure; (**c**) and (**d**) representative microstructure containing 180° domains [135].

Nevertheless, in recent years, a lot of research has been carried out in order to solve pure KNN processing problems and to improve the piezoelectric properties using two different strategies: changes in composition and/or changing the processing. Changes in composition while maintaining the versatile perovskite structure may be done either in the A site (*i.e*., by partially replacing sodium or potassium by lithium) or in the B site (partially replacing niobium by other elements such as tantalum, tungsten, antimony or others (Table 3). As a consequence of it, complicated KNN solid solutions are being prepared with compositions such as (K_0.44_Na_0.52_Li_0.04_)(Nb_0.86_Ta_0.10_Sb_0.04_)O_3_, prepared for the first time back in 2004 [118]. A peak piezoelectric constant *d*_33_ of 416 pC/N and a high converse piezoelectric coefficient (*d*_33_^*^ = S_max_/E_max_ ~ 750 pm/V) were obtained when preparing this compound in the textured polycrystalline form by using reactive templated grain growth (RTGG) methods. This was a major breakthrough and provoked enthusiasm for developing high-performance lead-free KNN-based piezoceramics. Although this special processing seemed to be crucial for these performances, meanwhile, aenhanced piezoelectricity (*d*_33_ = 200–235 pC/N) in KNN ceramics was also observed by doping LiNbO_3_ or LiTaO_3_ using conventional solid-state methods [128]. Thus, following different viewpoints, the research on KNN-based materials has been greatly accelerated. By substituting around 6% lithium into KNN, a MPB between orthorhombic and tetragonal phases has been found and high densities and piezoelectric properties (314 pC/N) can be obtained by conventional air sintering. Some authors make co-doping with Ta or Sb in the Nb position; sintering is conducted at 1060 °C [104,119,129]. The effects of stoichiometry and milling processes in the solid state synthesis and in the piezoelectric properties of these Li-Ta-Sb modified KNN materials (nano-sized) has been studied: the sintered ceramics are not hygroscopic and resistant to ambient conditions [130]. Interestingly enough, this kind of KNN material (modified or not) has been prepared by the “spray-drying” method, as an alternative to the ceramic method, yielding nano-sized powders which can be sintered to prepare ceramics of high density (96%) with improved properties [131,132]. With the same strategy, trying to avoid potassium or sodium losses by sublimation, these ceramics have been prepared by “fast chemistry” methods, such as spark-plasma synthesis (SPS) and microwave-assisted methods [133,134]. Nevertheless, regarding the control of grain growth, it is worth noting that self-orientation and agglomeration of nanoparticles can usually be observed besides microstructures consisting of domains and domain walls (Figure 8) [135], all these issues affecting the piezoelectric properties [136].

Table 3 summarizes the mentioned and some other relevant works [136,137,138,139,140,141,142,143,144,145,146,147,148,149,150,151,152,153,154] published on this solid solution series. Addition of nanoparticles (ZnO, CuO and SnO_2_) as sintering aids in Li-doped KNN materials have also been investigated but the piezoelectric coefficient *d*_33_ is decreased [137]. The effects of low-Li and high-Sb contents in the crystal structure of modified KNN based piezoceramics has been studied showing that the structure changes from tetragonal to pseudo-cubic. The ferroelectric Curie temperature shifts to lower temperature while the tetragonal to orthorhombic phase transition does not change [150]. On the contrary, rather than antimony, a “constructed quinquevalent cation” (W_2/3_Bi_1/3_) has been shown to be very effective in improving the electrical properties of KNN-based ceramics [148]. The addition of small amounts of WO_3_ changes the symmetry to monoclinic, induces an increase in the dielectric constant and a shift in the Curie temperature towards higher values as well [147]. In a similar approach, the effects of Mo^6+^ substitutions have been explored: up to 6% can be introduced in the Nb site without secondary phase’s segregation [152]. Other authors have investigated the effects of doping with silver in the dielectric properties of KNN-based ceramics prepared by templated grain growth [139].

Thus, plenty of work—in many directions—has been done on KNN since the first seminal paper [118] but the best results are quite recent and the replacement of PZT seems closer than ever. In this connection, “Giant Piezoelectricity” in an alkali niobate-based lead-free piezoceramic has been reported [153]. This KNN-based material shows the largest *d*_33_ of =490 pC/N ever reported so far using the conventional solid-state methodology. In addition, this material system also exhibits excellent integrated performance with *d*_33_ = 390−490 pC/N and *T*_C_ = 217–304 °C by optimizing the compositions. The authors ascribe these features to the construction of a new rhombohedral-tetragonal phase boundary. In the same direction of construction of new phase boundaries but with different compositions, the same research group has very recently reported excellent piezoelectric properties for materials of composition intermediate between two solid solutions: (1–*x*)(K_0.4_Na_0.6_)(Nb_0.96_Sb_0.04_)O_3_-*x*Bi_0.5_K_0.5_Zr_1__−_*_y_*Sn*_y_*O_3_ [154].

**Table 3:** Some results of the publications on compositions based on (K_0.5_Na_0.5_)NbO_3_.

Composition	Synthesis Method	Synthesis (*T (t)*) (°C (h))	Sintering (*T (t)*) (°C (h))	Grain Size/μm	ε^T^_33_/tan δ (%) (at RT)	*T*_C_/°C	*d*_33_/k_p_ pC/N/%	Reference
(K_0.5_Na_0.5_)NbO_3_	Sol-gel	650 (4)	1115 (2)	10	450/6	405	80/–	[138]
(K_0.5_Na_0.5_)NbO_3_	Solid state	825 (4)	1115 (2)	7	925/0.1	395	–/–	[139]
(K_0.5_Na_0.5_)NbO_3_	Spray drying	800 (1)	1080 (2)	10	380/2	410	–/36	[132]
(K_0.5_Na_0.5_)NbO_3_	Solid state	–	–	–	400/0.3	395	140/39	[6]
(K_0.5_Na_0.5_)NbO_3_	Solid state	850 (2)	1160 (2)	–	496/1.5	420	127/46	[120]
(K_0.5_Na_0.5_)NbO_3_	Solid state	850 (10)	1110 (-)	–	450/–	420	95/28	[128]
(K_0.5_Na_0.5_)NbO_3_	Sol-gel	500 (5)	925 (2)	2	200/4.8	405	112/20	[140]
(K_0.5_Na_0.5_)NbO_3_	Combustion	450 (6)	1120 (2)	1.45	490/2.8	420	84/–	[141]
(K_0.5_Na_0.5_)NbO_3_	Sol-gel	650 (2)	1110 (3)	5	712/6.7	405	90/32	[142]
(K_0.5_Na_0.5_)NbO_3_	Mechanochemical asisted	550 (2)	1100 (2)	0.3	680/3.5	420	95/–	[143]
(K_0.5_Na_0.5_)NbO_3_	Microwave-assisted	550 (2)	1115 (2)	3.8	427/3.5	398	85/–	[144]
(K_0.5_Na_0.5_)NbO_3_	Hydrothermal	230 (24)	1000 (2)	2	–/–	–	78/–	[145]
(K_0.5_Na_0.5_)_0.9_Li_0.1_NbO_3_	Sol-gel	500 (5)	975 (2)	2	400/2.8	485	170/40	[140]
(K_0.5_Na_0.5_)Nb_0.7_Ta_0.3_O_3_	Hydrothermal	230 (24)	1000 (2)	2	–/–	–	210/–	[145]
(K_0.5_Na_0.5_)NbO_3_-1%GeO_2_	Solid state	650 (2)	1000 (2)	5	397/2	–	120/40	[127]
(K_0.5_Na_0.5_)_0.99_Ca_0.05_NbO_3_	Sol-gel	650 (4)	1115 (2)	10	495/12	398	95/–	[138]
(K_0.5_Na_0.5_)_0.99_Sr_0.05_NbO_3_	Sol-gel	650 (4)	1115 (2)	10	500/4	397	95/–	[138]
(K_0.5_Na_0.5_)_0.94_Li_0.06_NbO_3_	Solid state	850 (4)	1060–1100 (2)	–	–/–	–	215/–	[146]
(K_0.17_Na_0.83_)NbO_3_ 5%WO_3_	Solid state	825 (4)	1160 (4)	5	1250/0.1	425	45/–	[147]
(K_0.5_Na_0.5_)_0.94_Li_0.06_NbO_3_	Solid state	850 (4)	1000 (2)	4	560/0.8	410	215/–	[137]
(K_0.48_Na_0.535_)_0.942_Li_0.058_NbO_3_	Solid state	750 (4)	1060 (2)	–	650/–	490	314/41	[151]
(K_0.5_Na_0.5_)_0.935_Li_0.065_NbO_3_	Solid state	800 (4)	1160 (1)	–	680/0.18	440	250/44	[119]
(K_0.5_Na_0.5_)NbO_3_-LiSbO_3_	Solid state	850 (2)	1200 (2)	–	1380/2	368	265/50	[120]
0.95(K_0.5_Na_0.5_)NbO_3_-0.05LiTaO_3_	Solid state	850 (10)	1110 (–)	–	570/4	425	200/36	[128]
(K_0.5_Na_0.5_)_0.948_(LiSb)_0.052_Nb_948_O_3_	Solid state	880 (4)	1080 (3)	–	1100/1.9	385	286/51	[129]
(K_0.5_Na_0.5_)_0.94_Li_0.06_(W_0.67_Bi_0.33_)_0.008_Nb_0.992_O_3_	Solid State	850 (4)	1100 (3)	7	950/2.5	–	282/45	[148]
0.92KNN-0.06BZ-0.02BLT	Molten salt & RTGG	800 (5)	1150 (2)	10	/–	251	271/–	[149]
(Na_0.5_K_0.5_)_0.975_Li_0.025_Nb_0.76_ Sb_0.06_Ta_0.18_O_3_	Solid state	890 (4.5)	1115 (3)	2.5	1000/2.5	200	352/47	[150]
(Na_0.44_K_0.515_Li_0.045_)Nb_0.915_ Sb_0.045_Ta_0.05_O_3_	Solid state	700 (6)	1100 (3)	40	1024/6.8	320	390/49	[136]
(Na_0.52_K_0.44_Li_0.04_)Nb_0.86_ Sb_0.04_Ta_0.1_O_3_	Solid state	700 (2)	1125 (16)	1.2	–/–	–	255/–	[130]
0.96(K_0.4_Na_0.6_)(Nb_0.96_Sb_0.04_)O_3_-0.04Bi_0.5_K_0.5_Zr_0.9_Sn_0.1_O_3_	Solid state	850 (6)	1095 (3)	4	2000/–	250	460/47	[154]
0.96(K_0.48_Na_0.52_)(Nb_0.97_Sb_0.03_)O_3_-0.04Bi_0.5_(Na_0.82_K_0.18_)_0.5_ZrO_3_	Solid state	850 (6)	1130 (3h)	–	2200/–	227	490/48	[153]

RTGG = Reactive Templated Grain Growth.

## 6. Conclusions

As it was shown for all the lead-free compositions for piezoelectric ceramics considered here, the synthesis route determines the homogeneity, morphology and grain size of the powders. Conventional solid state routes were compared with wet chemistry synthesis methods. As a general statement, we can say that these alternative routes, by using softer conditions, help in keeping adequate stoichiometry, avoiding the volatilization associated with many of the cations involved in these compositions (bismuth, alkaline metals, *etc*.). Many of these routes also employ non-hazardous raw materials as reactants (salts, oxides, *etc*.). Nevertheless, care must be taken when considering other reactants involved in the proposed routes for environmental principles.

**Figure 9:**
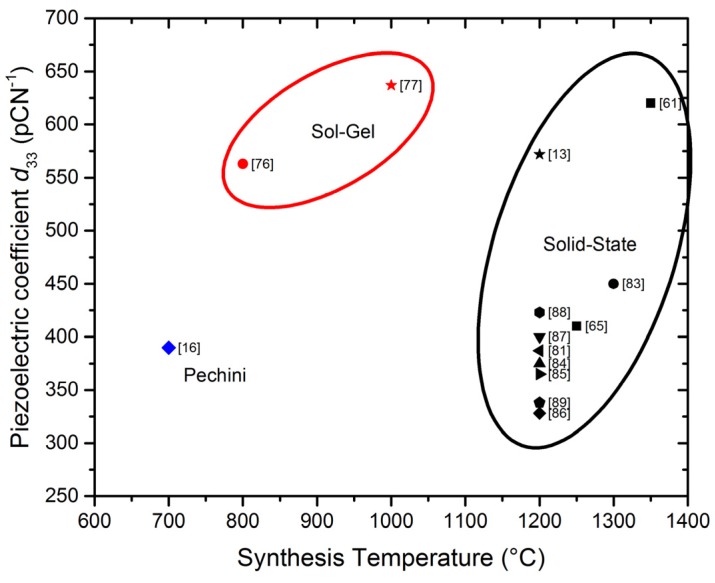
Piezoelectric coefficient *d*_33_ *vs*. synthesis temperature for the different methods of BCTZ ceramics preparation, extracted from references in Table 1.

**Figure 10:**
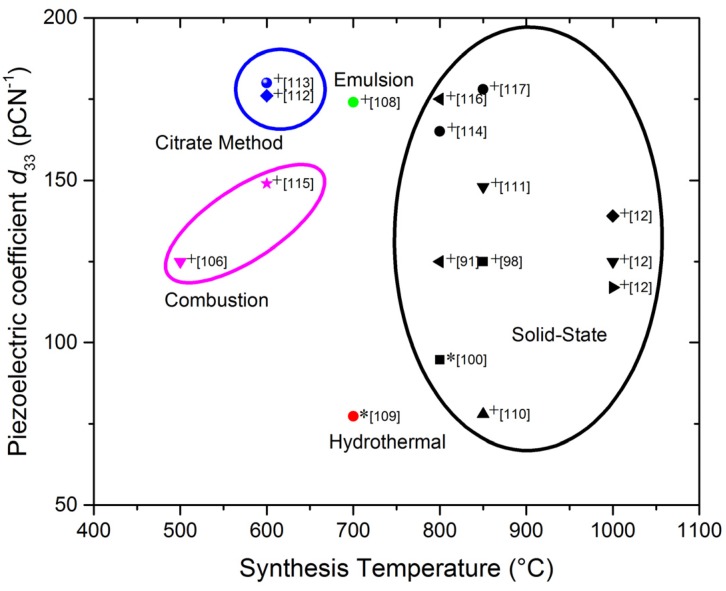
Piezoelectric coefficient *d*_33_ *vs*. synthesis temperature for the different methods of BNT-BT ceramics preparation, extracted from references in Table 2. (* Pure BNT. ^+^ BNT-based solid solution).

**Figure 11:**
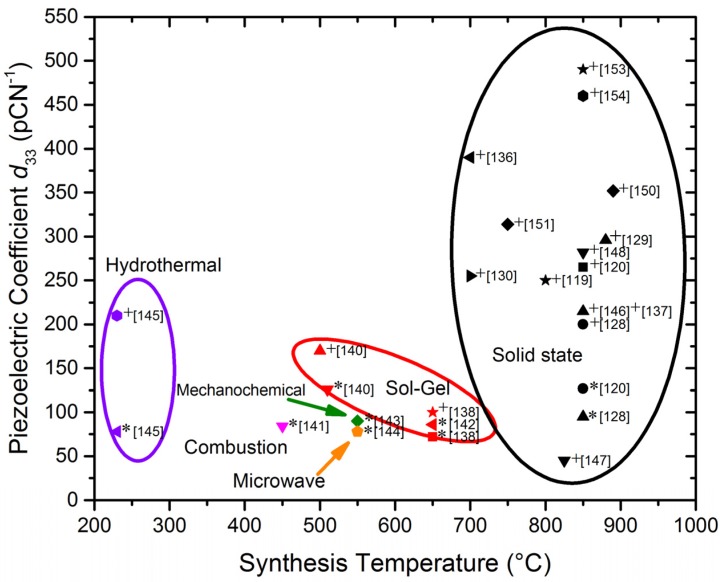
Piezoelectric coefficient *d*_33_ *vs*. synthesis temperature for the different methods of KNN ceramics preparation, extracted from references in Table 3. (* Pure KNN. ^+^ KNN-based solid solution).

In addition to these advantages, the use of lower synthesis temperatures is not of detriment to the final properties. Besides all these considerations, the small and homogeneous grain size promoted by solution methods enhances the sintering and densification of the bulk material, which favors the ceramic performance. Figure 9, Figure 10 and Figure 11 show the *d*_33_ coefficient *vs.* the synthesis temperature.

Figure 9 shows the *d*_33_ coefficient for BCTZ ceramics, extracted from references in Table 1. It is noticeable the wide range of *d*_33_ values for ceramics obtained by solid state synthesis, but always using synthesis temperatures from 1200 °C. The variation of the *d*_33_ values is explained by the composition studied as shown in the ternary diagram of Figure 6. Alternative routes were tested for the ceramics with better performance. For sol–gel methods, the *d*_33_ values obtained using synthesis temperatures below 1000 °C are among the highest.

Figure 10 shows the same analysis for BNT-BT based compositions, extracted from data in Table 2. Synthesis temperatures for all methods are lower than in BCTZ. Again, solid state synthesis requires higher temperatures, above 800 °C, with the risk of bismuth loss. Alternative methods allow obtaining a similar range of *d*_33_ values with synthesis temperatures below 700 °C.

The same analysis carried out in the KNN based ceramics is shown in Figure 11. To understand that the best *d*_33_ values were achieved for solid state synthesis, the strong effect of cation substitutions (see Table 3) on the properties of this solid solution must be considered. Recent work [153] obtained great piezoelectricity by compositional design. This makes it difficult to directly compare properties of different compositions. Focusing on the unmodified compositions, marked with an asterisk in Figure 11, we can arrive at the same conclusion as for previous solid solutions (Figure 9 and Figure 10).

For a given composition, an analysis of the best route according to the available reactants for each element must be carried out, since there is not a single recipe or universal procedure. Softer conditions also imply a more efficient use of energy with beneficial impact on the industrial transference.

Besides all these considerations, the small and homogeneous grain size promoted by solution methods enhances the sintering and densification of the bulk material, which favors the ceramic performance. For optimized sintering parameters, generally speaking, the adequate poling conditions—temperature, time and electric field—determine the properties of piezoelectric ceramics. Also, when considering lead-free ceramics, care must be taken regarding the crystallographic phase transitions among polymorphs induced by compositional changes, temperature and electric field, especially near MPBs.
